# Engineering Thermal Cross-Linking in Nanofiltration Membranes for Efficient Nicotine Extraction from Tobacco Extract

**DOI:** 10.3390/membranes15110327

**Published:** 2025-10-28

**Authors:** He Du, Xinyuan Wang, Baodan Na, Yajun Ye, Yuemei Qiao, Linda Li, Ye Tian, Xiaoping Ning, Zhigang Wang, Xingquan Zhao, Chen Chen

**Affiliations:** Inner Mongolia Kunming Cigarette Co., Ltd., Inner Mongolia, Hohhot 010020, China; mkjsyf@163.com (H.D.); 18847622296@163.com (X.W.); nabaodan929@163.com (B.N.); yajuntobacco@163.com (Y.Y.); l17704885884@163.com (L.L.); mktiany3039@163.com (Y.T.); 15849388358@163.com (X.N.); 13664786278@163.com (Z.W.); lhwz17@163.com (X.Z.)

**Keywords:** nanofiltration membrane, integrally asymmetric membrane, tobacco extract, molecular weight cut-off, thermal crosslinking

## Abstract

Tobacco extract contains numerous valuable components, among which nicotine possesses significant potential for high-value applications despite its well-known health risks. However, the efficient extraction of nicotine is challenging due to the complex composition of tobacco extracts and the limitations of conventional separation techniques. In this work, an integrally asymmetric nanofiltration membrane was developed via thermal cross-linking for highly efficient nicotine separation. A poly(aryl ether ketone) (PEK)-based ultrafiltration membrane was first prepared via non-solvent induced phase separation (NIPS), followed by controlled thermal cross-linking to tailor the membrane pore size toward the molecular weight of nicotine. To mitigate pore collapse and enhance flux, TiO_2_ nanoparticles were incorporated in situ through a sol–gel method. The resulting thermally cross-linked membrane exhibited a molecular weight cut-off of ~180 Da, a nicotine rejection rate of 93.2%, and a permeation flux of 143 L/(m^2^·h)—representing a 259% increase over the control membrane. Moreover, the thermally cross-linked membranes demonstrated exceptional chemical stability in various organic solvents and extreme pH conditions. This work offers a feasible and sustainable strategy for fabric high-performance nanofiltration membranes for the targeted extraction of bioactive molecules from complex plant extracts.

## 1. Introduction

Tobacco extract contains a substantial amount of nicotine, a compound widely recognized for its adverse effects on human health. Nevertheless, nicotine also possesses significant potential for high-value applications due to its role as a precursor in pharmaceuticals, pesticides, and other fine chemicals [1,2]. The precise separation of active components—especially nicotine—from tobacco extracts is essential for enhancing the value-added utilization of tobacco crops, promoting the transformation and upgrading of the tobacco industry, and achieving green, efficient, and sustainable development [3,4]. Tobacco extracts are typically obtained through solvent extraction of dried tobacco leaves, resulting in a complex mixture containing nicotine, sugars, organic acids, polyphenols, alkaloids, and inorganic salts [4]. These components exhibit a wide molecular weight distribution (∼50–500 Da) and varying chemical properties, making separation challenging. Conventional separation techniques such as distillation, extraction, chromatography, adsorption, and crystallization often suffer from low efficiency and high energy consumption, highlighting the urgent need for developing novel technologies for efficient nicotine separation [5,6,7,8,9,10].

Nanofiltration (NF), a membrane-based separation technology, has shown great promise for the selective separation of organic molecules within the 200–1000 Da range [11,12,13]. This process offers notable advantages, including low energy consumption, high separation accuracy, solvent-free operation, and the absence of phase transitions, thereby preserving the bioactivity of target compounds in tobacco extracts [8,14,15]. In industrial practice, NF is envisioned as a downstream purification step following preliminary coarse separation (e.g., ultrafiltration or adsorption) to selectively concentrate nicotine while removing smaller impurities and larger macromolecules. The core of NF technology lies in the nanofiltration membrane, whose performance is governed by the synergistic effects of size exclusion (controlled by sub-nanometer pores), Donnan exclusion (influenced by surface charge), and non-electrostatic adsorption (determined by surface chemical properties) [16,17,18]. Accordingly, it is feasible to tailor NF membranes with specific molecular weight cut-offs (MWCOs) and surface characteristics through advanced fabrication and modification strategies to achieve precise extraction or enrichment of target molecules.

Most commercially available nanofiltration membranes are thin-film composite (TFC) membranes fabricated via interfacial polymerization on an ultrafiltration substrate [19,20,21,22,23,24,25], such as the Dow Filmtec™ NF series, Toray’s TNF series, and Hydranautics’ ESNA series. While widely used, these membranes often exhibit limited permeance and poor chemical stability, rendering them less suitable for treating complex solutions such as tobacco extracts [18,26,27,28]. While NF technology has been widely applied in bio-separation and solvent-resistant nanofiltration, its specific application for nicotine extraction from tobacco extracts remains relatively underexplored. This work presents the development of precisely tailored integrally asymmetric NF membranes via thermal cross-linking for high-efficiency nicotine separation. The process involves first preparing an ultrafiltration membrane via phase inversion, followed by controlled thermal cross-linking treatment to induce pore narrowing, thereby tuning the membrane’s effective pore size to match the molecular dimension of nicotine for efficient rejection. Furthermore, to maintain high permeance while achieving selectivity, titanium dioxide (TiO_2_) nanoparticles were incorporated into membranes in situ via a sol–gel method, which effectively suppress pore collapse and coalescence during thermal shrinkage. The resulting thermally cross-linked nanofiltration membrane demonstrates high permeance and rejection performance toward nicotine-containing solutions, alongside remarkable stability in various chemical environments—including alkanes, alcohols, ketones, esters, aromatic compounds, acids, and bases—such as those encountered in tobacco extract processing. These attributes underscore its strong potential for practical application in the efficient and sustainable extraction of nicotine.

## 2. Experimental Methods

### 2.1. Materials

The polymer material used for membrane preparation, poly(aryl ether ketone) (PEK), was supplied by Xuzhou Aviation Engineering Plastics Co., Ltd. (Mw = 217,000, *T*_g_ = 240 °C, Xuzhou, China). The solvent for the casting solution, *N*,*N*-dimethylacetamide (DMAc), was purchased from Tianjin Damao Chemical Reagent Factory (Tianjin, China). Tetrabutyl titanate (TBT) and glacial acetic acid were obtained from Tianjin Fuchen Chemical Reagent Co., Ltd. (Tianjin, China). Bovine serum albumin (BSA, molecular weight = 68 kDa) was procured from Beijing Probeene Biotechnology Co., Ltd. (Beijing, China). Analytical-grade saccharides, including raffinose, sucrose, glucose, and glycerol, were provided by Sinopharm Chemical Reagent Co., Ltd. (Shanghai, China). Chemical reagents such as n-hexane, isopropanol, acetone, ethyl acetate, toluene, sodium hydroxide (NaOH), and hydrochloric acid (HCl) were acquired from Tianjin Fuyu Fine Chemical Co., Ltd. (Tianjin, China).

### 2.2. Preparation of Membranes

The dried PEK powder was dissolved in DMAc organic solvent to prepare a casting solution with a concentration of 16 wt%. The mixture was stirred and heated at 80 °C for 6 h to ensure complete dissolution of the polymer. After cooling to room temperature, the solution was subjected to vacuum degassing and then stored undisturbed for subsequent membrane casting. TiO_2_ nanoparticles were introduced in situ into the PEK polymer matrix via a sol–gel method. Specifically, 2 g of glacial acetic acid was added to DMAc solvent under stirring. A predetermined amount of TBT was then added rapidly under vigorous shaking to prevent hydrolysis and aggregation, forming a transparent and homogeneous sol. This sol was added dropwise into the 16 wt% PEK casting solution under rapid stirring. After complete addition, stirring was continued for 2 h, followed by final vacuum degassing. The amount of TBT was controlled at 25 wt% relative to the mass of PEK. Flat-sheet membranes were fabricated via the non-solvent induced phase separation (NIPS) method. The casting solution without TBT doping was cast using deionized water as the coagulation bath, while the TBT-doped casting solution was coagulated in a hydrochloric acid bath (pH = 2) maintained at 60 °C. The resulting membranes were dried in a vacuum oven at 60 °C for 12 h to obtain the ultrafiltration membranes, designated as M_0_ and M_1_, respectively.

Thermal crosslinking treatment: the M_0_ and M_1_ membranes were placed in a muffle furnace under a continuous flow of dry air at a rate of 2 L/min. The temperature was first increased from room temperature to 100 °C at a heating rate of 5 °C/min, then raised to 200 °C at 3 °C/min, and finally elevated to 280 °C at 0.5 °C/min. The membranes were maintained at 280 °C for 5 h, followed by natural cooling to room temperature. The resulting thermally cross-linked membranes were designated as TM_0_ and TM_1_, respectively. The compositions of membranes as well as their fabricating conditions were listed in Table 1.

### 2.3. Characterizations

The surface and cross-sectional morphologies of the prepared membranes were examined using a QUANTA450 scanning electron microscope (FEI Company, Hillsboro, OR, USA). The membrane samples were cryogenically fractured in liquid nitrogen to obtain clean cross-sections without deformation, then vacuum-dried and sputter-coated with a thin gold layer prior to imaging. The thermal decomposition behavior of the membranes was evaluated using a STA 209-F1 thermogravimetric analyzer (Netzsch Company, Waldkraiburg, Germany) under a nitrogen atmosphere. The heating rate was set at 10 °C/min with a nitrogen flow rate of 20 mL/min. The hydrophilicity of the membranes was evaluated by measuring the pure water contact angle using a JC200D contact angle goniometer (Shanghai Zhongchen Digital Technology Equipment Co., Ltd., Shanghai, China). A 2 μL water droplet was used at room temperature using the sessile drop method. The measurements were repeated at least five times at different locations on each membrane surface to ensure statistical reliability, and the average value was reported. The water contact angles were measured and processed with the JC200D system’s built-in analysis software. Surface zeta potentials of membranes at varying pH levels (3.0–9.0) were determined via the streaming potential method on a SurPASS 3 electrokinetic analyzer (Anton Paar GmbH, Graz, Austria).

### 2.4. Measurements of Separation Properties

The permeation flux and rejection performance of the membranes were evaluated using a dead-end filtration cell. The membrane was cut into circular samples with a diameter of 2.5 cm and placed in the membrane cells. The membranes were firstly pre-compacted at 0.5 MPa for 30 min until flux stabilization. The flux and rejection were measured under an operating pressure of 0.4 MPa. The flux was calculated using Equation (1):

(1) $$F=\frac{Q}{At}$$

where *F* represents the flux (L/(m^2^·h)), *Q* is the volume of permeate (L), *A* denotes the effective membrane area (m^2^), and *t* refers to the filtration duration (h).

The rejection rates (*R*) of the test substances were calculated using the following Equation (2). The target molecules evaluated in this study included BSA, probe molecules such as raffinose, sucrose, glucose, and glycerol, as well as nicotine. All solutions were prepared at a concentration of 1 g/L. The concentration of permeate solution for nicotine was analyzed by gas chromatography–mass spectrometry (GC-MS), while other concentrations were measured using a UV-vis spectrophotometer.

(2) $$R=(1-\frac{C_{p}}{C_{f}})\times 100\%$$

where *C_p_* and *C_f_* represent the concentrations (g/L) of the permeate and feed solutions, respectively.

## 3. Results and Discussion

### 3.1. Properties of UF Membranes

**Figure 1:**
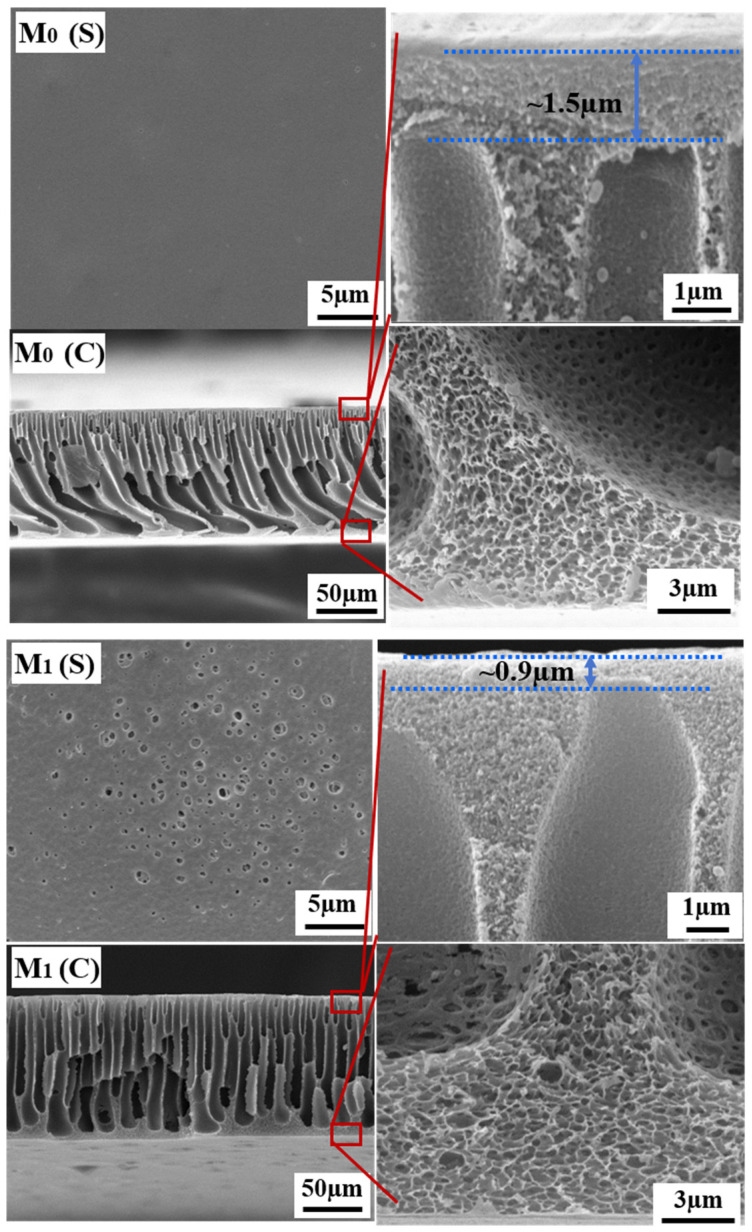
Surface and cross-sectional SEM images of the M_0_ and M_1_ UF membranes.

**Figure 2:**
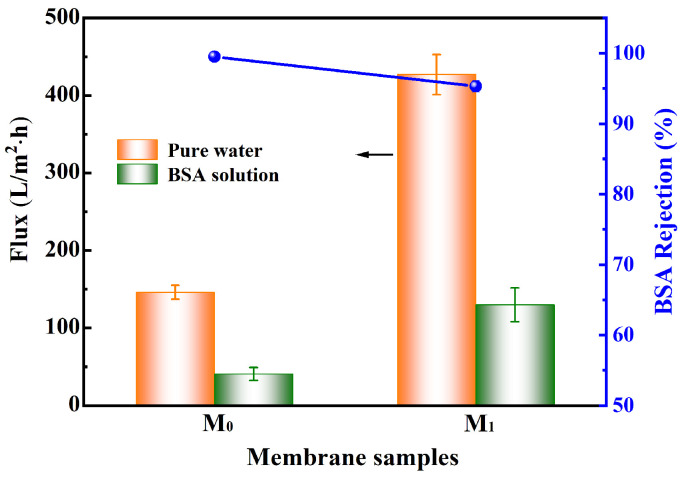
Pure water and BSA solution flux and BSA rejection of the M_0_ and M_1_ UF membranes.

Figure 1 presents the surface and cross-sectional SEM images of the PEK-based ultrafiltration membranes prepared via NIPS, revealing a typical asymmetric structure consisting of a selective skin layer, sponge-like pores, and finger-like macrovoids. The M_0_ membrane exhibits a smooth surface without visible pores at this resolution. Its cross-section shows a dense skin layer approximately 1.5 μm in thickness, accompanied by interconnected finger-like pores and highly porous sponge-like regions between the macrovoids. In contrast, the M_1_ membrane, fabricated with the introduction of TBT as a titanium source, underwent in situ sol–gel reactions during phase inversion, leading to the formation of TiO_2_ nanoparticles within the polymer matrix. Partial segregation and migration of the nanoparticles likely resulted in the formation of larger surface pores. Cross-sectional analysis indicates a significantly reduced skin layer thickness of about 0.9 μm, along with open finger-like channels and more loosely structured sponge-like pores. These structural characteristics are conducive to enhancing membrane permeance and facilitating air penetration during subsequent thermal cross-linking, thereby promoting adequate oxidative cross-linking reactions. Consistent with the alterations in membrane pore structure, Figure 2 demonstrates that the M_1_ membrane exhibits a significant improvement in both pure water flux and BSA solution flux compared to the M_0_ membrane. The pure water flux increased from 146 L/m^2^·h to 427 L/m^2^·h, while the BSA rejection rate correspondingly decreased from 99.5% to 95.3%.

**Figure 3:**
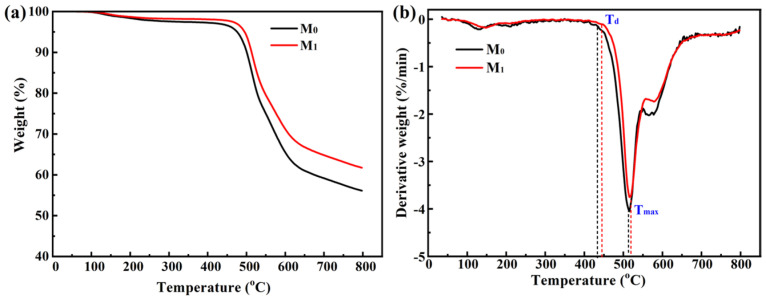
(**a**) TG and (**b**) DTG curves of the M_0_ and M_1_ UF membranes in N_2_.

The TG and DTG curves of M_0_ and M_1_ membranes (Figure 3) reveal that the thermal decomposition temperatures—including the initial decomposition temperature (*T*_d_) and the temperature at maximum decomposition rate (*T*_max_)—of the M_1_ membrane are slightly higher than those of the pristine M_0_ membrane, indicating enhanced thermal stability. This improvement may be attributed to the in situ formed TiO_2_ cross-linked network via the sol–gel method, which strengthens the intermolecular interactions within the polymer matrix. Additionally, the hydroxyl groups on the surface of TiO_2_ nanoparticles can form hydrogen bonds with functional groups in the polymer chains, further enhancing intermolecular forces and thermal stability. This effect helps mitigate polymer chain melting during the thermal crosslinking treatment process, thereby contributing to the preservation of the membrane’s porous structure.

### 3.2. Properties of Thermal Crosslinking Membranes

**Figure 4:**
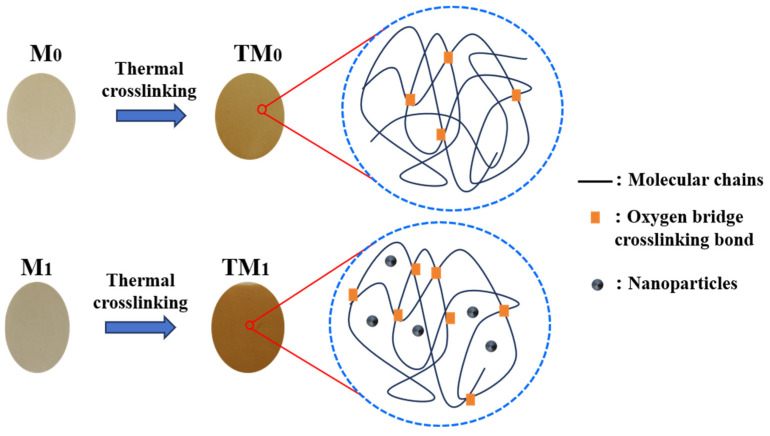
Color changes in the M_0_ and M_1_ membranes after thermal crosslinking treatment and schematic diagram of thermal crosslinking molecular structure.

Previous studies have reported that when the heat treatment temperature exceeds 250 °C in an air atmosphere, oxidative cross-linking occurs between PEK polymeric chains [29,30,31], leading to the formation of oxygen-bridged bonds, as illustrated in Figure 4. A stable cross-linked network is thus established, which enhances the chemical stability of the membrane—particularly its resistance to organic solvents. Macroscopically, after thermal cross-linking, the membrane surface changes color from white to yellowish-brown, accompanied by observable thermal shrinkage. Compared to the M_0_ membrane, the M_1_ membrane containing TiO_2_ nanoparticles also undergoes oxidative cross-linking. Owing to its more developed porous structure, the M_1_ membrane facilitates enhanced air penetration, resulting in a higher degree of cross-linking. Accordingly, the resulting TM_1_ membrane exhibits a darker color. Furthermore, the presence of TiO_2_ nanoparticles strengthens the interactions between the polymeric chains, improving the thermal resistance of the membrane and reducing thermal shrinkage [32]. This effect also contributes to maintaining the original porous structure of the nascent membrane. The surface and cross-sectional SEM images of the thermally cross-linked membranes provide further evidence supporting these conclusions.

**Figure 5:**
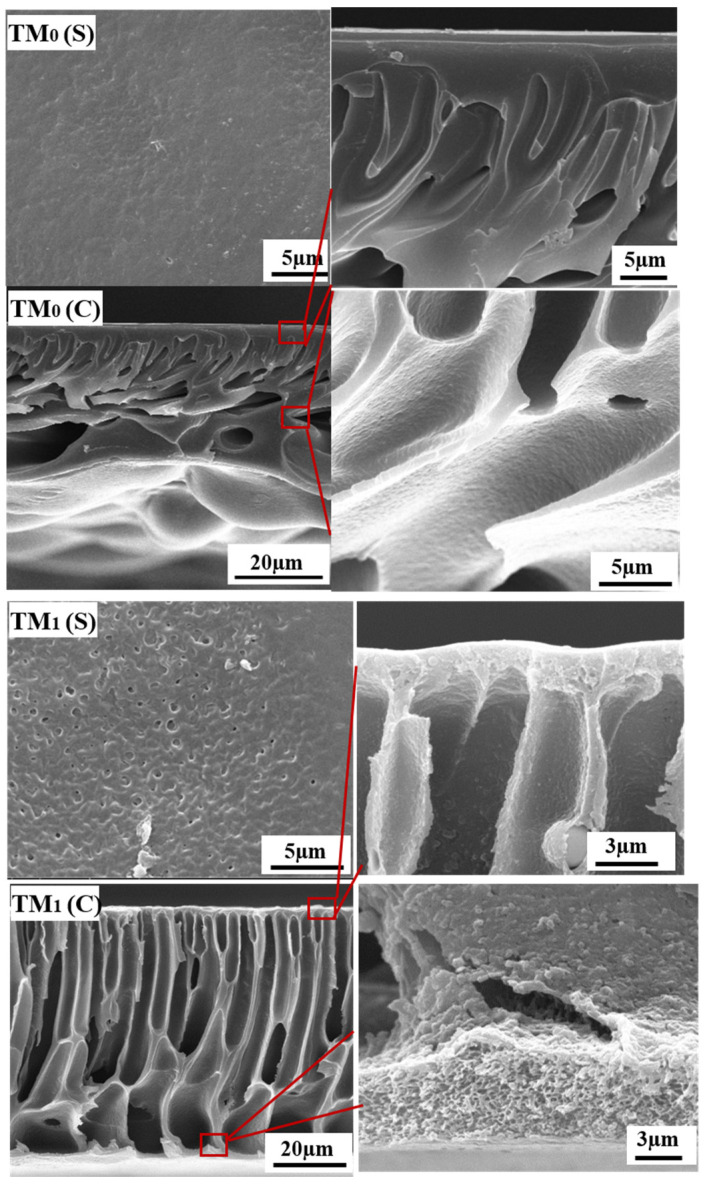
Surface and cross-sectional SEM images of the TM_0_ and TM_1_ thermal crosslinking membranes.

Figure 5 shows that after thermal cross-linking treatment, the combined effects of thermal shrinkage and oxidative cross-linking result in a denser surface layer of the TM_0_ membrane. Cross-sectional observations reveal that although an asymmetric pore structure is still present, the sponge-like pores have largely merged and disappeared. The thickness of the dense skin layer increased significantly, and the sponge-like pores between the finger-like pores were also eliminated. In contrast, the TM_1_ membrane exhibited significantly reduced thermal shrinkage. Although pore fusion and densification of the surface still occur due to heating, the cross-sectional images clearly show diminished thermal shrinkage. The skin layer of TM_1_ is noticeably thinner than that of TM_0_, and a small number of sponge-like pores remain near the bottom surface and the walls of the finger-like pores. This preserved porous structure is expected to contribute positively to the membrane’s permeance.

**Figure 6:**
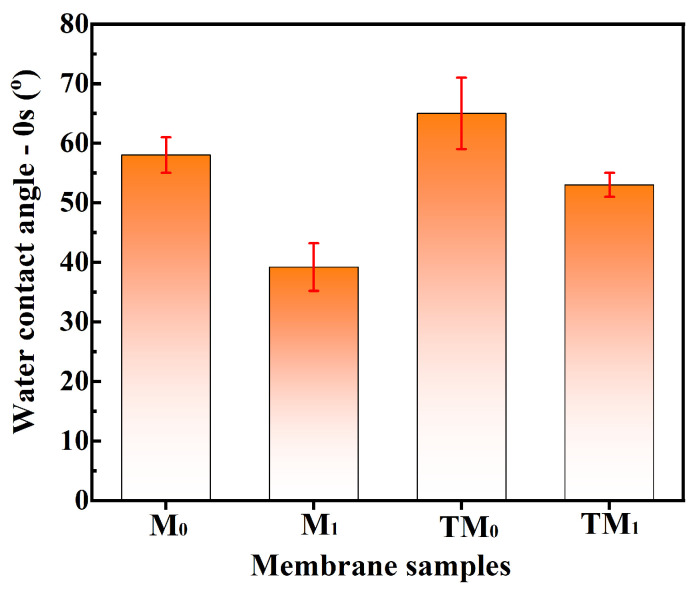
Water contact angle at 0 s of membranes.

**Figure 7:**
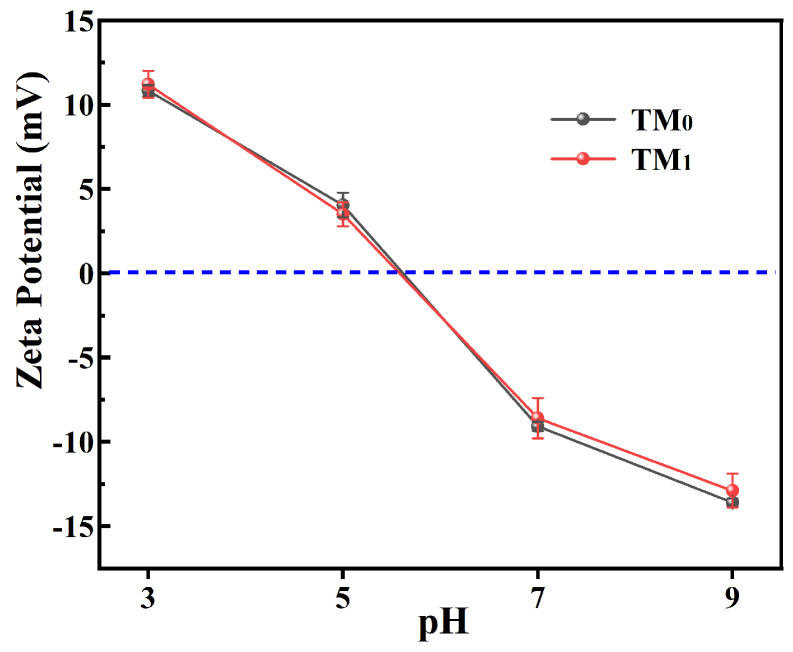
Zeta potential at pH (3~9) of membranes.

Figure 6 shows the water contact angles at 0 s of the membranes. All obtained membranes exhibited contact angles of less than 90°, indicating good hydrophilicity. Compared to the M_0_ membrane, the M_1_ membrane showed a lower contact angle, which is primarily attributed to the incorporation of hydrophilic TiO_2_ nanoparticles. After thermal cross-linking, the surface pores contracted, leading to densification of the skin layer and a slight increase in the water contact angle. Nevertheless, the contact angle of TM_1_ remained lower than that of TM_0_, which also contributes to enhancing the permeation flux of the membrane. The zeta potential of the TM_0_ and TM_1_ thermal crosslinking membranes under different pH conditions indicates that the membrane surfaces are positively charged at pH below 5.5 and negatively charged at pH above 5.5 (Figure 7). This charge characteristic facilitates the rejection of negatively charged components or anions in tobacco extract through Donnan exclusion effects [14]. Given that nicotine is predominantly uncharged under the experimental conditions of tobacco extract processing, the Donnan exclusion effect might have played a limited role in the current study. In subsequent industrial applications, adjusting the tobacco extract to acidic conditions (e.g., pH < 5) may further enhance nicotine rejection by promoting its protonation and leveraging charge-based repulsion mechanisms.

**Figure 8:**
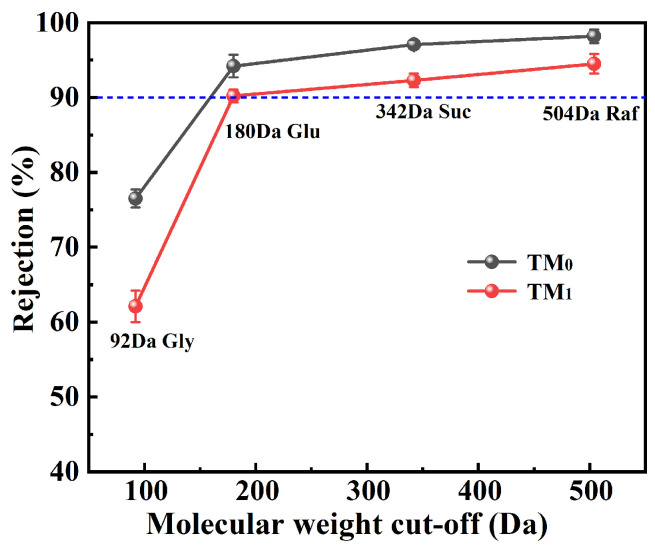
Molecular weight cut-off of membranes.

To evaluate the molecular weight cut-off (MWCO) of the TM_0_ and TM_1_ thermal crosslinking membranes, four neutral organic solutes with different molecular weights were selected as probe molecules: raffinose (Raf, 504 Da), sucrose (Suc, 342 Da), glucose (Glu, 180 Da), and glycerol (Gly, 92 Da). The rejection rates of these molecules by the membranes were measured accordingly. As shown in Figure 8, the rejection rate increases with the molecular weight of the probe molecules, demonstrating a clear molecular sieving effect. Specifically, for each probe molecule, the TM_0_ membrane exhibited higher rejection rates than the TM_1_ membrane, which is closely related to their pore structures and indirectly confirms that the average pore size of TM_1_ is slightly larger than that of TM_0_. Furthermore, both TM_0_ and TM_1_ membranes showed rejection rates exceeding 90% for glucose. According to the standard definition of MWCO [33], their MWCO can be determined as 180 Da. This value matches the molecular weight of nicotine (162 Da) in tobacco extract, indicating that the membranes are suitable for the extraction and concentration of nicotine from tobacco extracts.

### 3.3. Separation Performance Toward Nicotine

**Figure 9:**
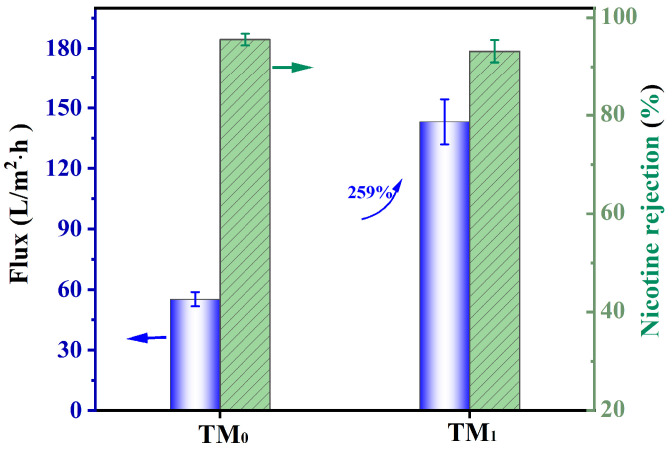
Flux and rejection of TM_0_ and TM_1_ membranes for nicotine solution.

The flux and nicotine rejection performance of the as-prepared TM_0_ and TM_1_ thermal crosslinking membranes were evaluated using an ethanol solution containing nicotine at a concentration of 1 g/L, as shown in Figure 9. Owing to the MWCO of the membranes being approximately 180 Da, which corresponds closely to the molecular weight of nicotine (162 Da), both TM_0_ and TM_1_ membranes exhibited high nicotine rejection rates of 95.6% and 93.2%, respectively, based on the molecular sieving mechanism. Notably, the TM_1_ membrane demonstrated a significantly higher permeation flux—an increase of 259% compared to TM_0_—rising from 55.2 L/(m^2^·h) to 143 L/(m^2^·h). This considerable improvement in flux can be attributed to the enhanced surface hydrophilicity, thinner separation skin layer, and retained sponge-like pore structure of the TM_1_ membrane. Therefore, the in situ incorporation of TiO_2_ nanoparticles via the sol–gel method is an effective strategy for enhancing the permeation flux of thermal crosslinking membranes via enhancing water transport pathways. The TM_1_ membrane also demonstrated excellent short-term operational stability during a continuous 10-h filtration test at 0.4 MPa, with the permeation flux decreasing by ~8% and the nicotine retention rate increasing by about 3%.

The TM_1_ membranes demonstrated excellent permeation flux and nicotine rejection performance in a model nicotine solution system, however, its performance may be influenced by competitive adsorption, fouling, and multi-solute interactions when treating real tobacco extracts. Future work should focus on applying this membrane to real tobacco extracts, where the NF step could be employed following preliminary clarification steps (e.g., via microfiltration/ultrafiltration) to remove suspended solids and macromolecules. The NF process would subsequently concentrate nicotine while partially removing solvents and salts. The resulting nicotine-rich retentate may undergo further purification through chromatographic techniques or additional tailored NF processes. Such an integrated membrane-based strategy shows significant potential for efficient nicotine extraction and concentration, thereby facilitating the high-value utilization of tobacco-derived compounds.

### 3.4. Stability of Thermal Crosslinking Membrane

The TM_1_ thermal crosslinking membrane was immersed in various chemical reagents for 10 days, including n-hexane, isopropanol, acetone, ethyl acetate, toluene, 1 mol/L NaOH, and 1 mol/L HCl. The permeation flux and nicotine rejection performance of the membrane were then evaluated, and the changes in these properties compared to the initial performance were summarized in Table 2. It can be observed that both the flux and nicotine rejection rate of the TM_1_ membrane exhibited only minor changes, indicating excellent chemical resistance and suitability for practical applications. These results demonstrate that the TM_1_ membrane developed in this study can maintain stable performance over extended periods in complex solvent environments—including alkanes, alcohols, ketones, esters, aromatic compounds, acids, and bases—such as those encountered in tobacco extract processing, demonstrating promising potential for real-world use.

## 4. Conclusions

An integrally asymmetric nanofiltration membrane was successfully fabricated through a thermal cross-linking strategy for highly efficient nicotine separation from tobacco extract. The incorporation of TiO_2_ nanoparticles via sol–gel method effectively inhibited pore coalescence during thermal treatment, resulting in a membrane with optimized pore structure, enhanced surface hydrophilicity, and reduced skin layer thickness. The tailored TM_1_ membrane showed a precise MWCO of 180 Da, which closely matches the molecular weight of nicotine, enabling high rejection (93.2%) alongside significantly improved permeation flux. Furthermore, the TM_1_ membrane exhibited remarkable stability in a wide range of chemical environments, including organic solvents and aggressive media, confirming its robustness for real-world applications. Unlike many TFC membranes, the membrane combines high flux and excellent chemical resistance without requiring additional post-modification. This study provides a feasible and green approach to designing high-performance nanofiltration membranes for the selective recovery of high-value components from complex biomass extracts.

## Figures and Tables

**Table 1:** Compositions of membranes as well as their fabricating conditions.

Fabricating Conditions	M_0_	M_1_
Polymer type and concentration	16 wt% PEK	16 wt% PEK
Amount of nanoparticle additive	without	25 wt% TBT
Coagulation bath composition and temperature	deionized water, 25 °C	hydrochloric acid bath (pH = 2), 60 °C
Thickness	95~105 μm	120~130 μm
Thermal cross-linking conditions	280 °C for 5 h	280 °C for 5 h
Thermally cross-linked membranes and thickness	TM_0_ 30~40 μm	TM_1_ 70~85 μm

**Table 2:** The changes in permeation flux and nicotine rejection rates of the TM_1_ membrane after being soaked in different solvents.

Types of Solvent	Flux Change Rate (%)	Rejection Change Rate (%)
n-hexane	4.30	−1.2
isopropanol	1.20	−0.86
acetone	−1.21	1.78
ethyl acetate	−2.24	1.53
toluene	1.56	−2.22
1 mol/L HCl	1.45	−0.83
1 mol/L NaOH	−2.14	1.11

## Data Availability

Data available on request from the authors.
